# Practical Utility of Diagnostic Clinical Breast Examination in the Diagnosis of Breast Cancer

**DOI:** 10.7759/cureus.17662

**Published:** 2021-09-02

**Authors:** Muberra Turan, Fisun Sozen, Muzaffer G Eminsoy, Tugce Sencelikel, Altug Kut, Sedat Yildirim, Ergun Oksuz

**Affiliations:** 1 Family Medicine, Baskent University Faculty of Medicine, Ankara, TUR; 2 Biostatistics, Baskent University Faculty of Medicine, Ankara, TUR; 3 General Surgery, Baskent University Faculty of Medicine, Ankara, TUR

**Keywords:** physical examination, breast neoplasms, breast diseases, mammography, breast ultrasonography

## Abstract

Objectives

We aimed to investigate the effectiveness of physician-performed diagnostic clinical breast examination (DCBE) for the diagnosis of breast cancer in clinical practice and to determine the rates of breast cancer diagnosed with DCBE compared to the results of breast ultrasonography (US), mammography (MG), and histopathology.

Methods

In the retrospective cohort study, the files of female patients diagnosed with breast cancer and admitted to the general surgery outpatient clinics of a university hospital over a 10-year period (2011-2021) were examined. Patients with complete DCBE findings in their files were identified and analyzed (n = 1,091). The examinations of the patients were performed by general surgery specialists with 5-22 years of experience and by radiologists with 4-15 years of experience.

Results

The mean age of breast cancer diagnosis of the patients was 55.1 ± 13.5 years. While the sensitivity of DCBE was found to be 88.9%, MG sensitivity was 89.8% and breast US sensitivity was 95.1%. Cancer was detected by MG, breast US, and DCBE in 47.9% (n = 523), by breast US and DCBE in 38.9% (n = 424), by MG and breast US in 5.6% (n = 61), by DCBE alone in 3.6% (n = 39), by MG and DCBE in 2.4% (n = 26), and by breast US alone in 1.6% (n = 18). Early-stage breast cancer (p = 0.00) consisted of 73.2% (n = 383) of cancers detected with DCBE, breast US and MG, 74.6% (n = 316) of cancers detected with DCBE and breast US, 93.4% of cancers detected with breast US and MG (n = 57), 92.3% (n = 24) of cancers detected with DCBE and MG, 94.4% (n = 17) of cancers detected with breast US alone, and 69.2% of cancers detected with DCBE alone (n = 27).

Conclusions

CBE still maintains its importance in societies where screening participation and awareness of breast cancer are low. A breast cancer diagnosis is often done after a complaint of a palpable mass in the breast, and only then are more advanced-stage breast cancers are seen. CBE is among the important diagnostic methods preventing breast cancer from being overlooked, especially in places where health resources are limited.

## Introduction

Breast cancer is the most common malignancy in women under 60 years of age worldwide and is one of the major causes of morbidity and mortality [1]. One out of every eight women is likely to develop breast cancer at some point in her life [2,3], and it is estimated that one out of every 33 women will die from breast cancer [3]. The incidence of breast cancer in Turkey in 2020 was 46 per 100,000 women. Breast cancer constitutes 24% of all newly diagnosed cancers in women. Breast cancer ranks first among cancers diagnosed in women and second among cancers diagnosed across the sexes [4,5]. In Turkey, 53.9% of diagnosed breast cancers have metastasized [5]. In addition, unlike in the rest of the world, breast cancer tends to be seen at younger ages in Turkey, where 40% of women diagnosed with breast cancer in 2020 were in the 25-49 years age group [4].

Diagnosis of breast cancer is made by history, physical examination, breast imaging methods, and biopsy. Painless palpable mass, suspicious MG results, nipple discharge require clinical investigation of the patient. Regarding the patient’s medical history, previous breast diseases, positive family history, menstrual patterns, age of menarche, oral contraceptive (OCS) use, hormone replacement therapy (HRT), number of children, and breastfeeding duration should be asked. In the anamnesis, questions should also be asked about when the mass was noticed, the location of the pain, its localization, and its relationship with menstruation [6]. However, MG should be performed in postmenopausal women where the mass persists, and malignancy is suspected. Parenchymal deformities are an important finding. Breast ultrasonography (US) is used to differentiate the mass from cystic or solid masses [7,8].

Screening mammography (SMG) has been shown to reduce breast-cancer-specific mortality [2]. However, evidence for clinical breast examination (CBE) in screening is not sufficient [9-13]. CBE is an application that provides an opportunity to educate people about the importance of early diagnosis, breast cancer risks, and breast diseases [2,9,11]. Not all breast cancers can be detected with SMG, which is not recommended for women in certain age groups [11,14]. Many guidelines have excluded the SCBE from their recommendations [15-19].

The aim of this study is to determine the efficiency of physician-performed DCBE for the diagnosis of breast cancer in routine clinical practice and to determine the rates of breast cancer diagnosed with diagnostic CBE (DCBE) alone.

## Materials and methods

In this retrospective cohort study, the files of patients who applied to a university hospital's general surgery outpatient clinic for breast examination between January 2011 and December 2021 were investigated. Female patients diagnosed with breast cancer were included in the study. For this purpose, female patients with the International Classification of Diseases (ICD-10) diagnostic codes C50 and D05 in their hospital files were selected (N=2,912). Of the 2,912 patients, 1,821 files (62.5%) who did not met study criteria were excluded; 48%; n=1,396 incomplete examination information, 8%; 233 the first diagnosis was made in another hospital, 5%; n=134 incomplete pathology results, and 2%; n=58 the definitive diagnosis was lobular carcinoma in situ (LCIS). Thus, among the patient records, 1,091 patients (37.5%; power=0.83, p= 0.034) who met the study criteria were identified and included in the analysis. During the research period, DCBE information recorded in the patients’ files during the examinations of six physicians who were experts in the field of general surgery in these polyclinics was used. These physicians had 5-22 years of medical experience.

Anamnesis in the patient files was examined, and the patients’ complaint at admission, age at diagnosis, age at menarche, age at menopause, age at first delivery, duration of lactation, use of OCS and/or HRT, history of breast disease, and family history of breast cancer were recorded. DCBE result: examinations with signs of a palpable mass, nodularity, nipple discharge, skin or nipple retraction, edema, discoloration, skin ulcer, axillary lymphadenomegaly, upper arm swelling, and tenderness were considered abnormal DCBEs (positive). Abnormal MG and/or breast US results were coded based on the radiology interpretation, and tumor information was coded based on the histopathology report.

Statistics

Research data were drawn from the hospital registry database with SQL (Structured Query Language) query. Patient files were coded in the computer environment, and the data were entered into IBM® (International Business Machines) SPSS® (Statistical Product and Service Solutions) Statistics 24.0 program (IBM Corp, Armonk, NY, USA) and analyzed through the program. Descriptive statistics for numerical variables were presented as mean (±) standard deviation, minimum and maximum values, and categorical data as frequency (n) and percentage (%). The conformity of the variables to the normal distribution was examined using visual (histogram and probability graphs) and analytical methods (Kolmogorov-Smirnov test if n ≥ 50, Shapiro-Wilk test if n < 50). In analyzing categorical data, depending on the assumptions, the significance of the 2% difference was evaluated with the Z-Test, chi-square test, or Fisher's exact test. The Student's t-test was used in the analysis of numerical variables. A value of <0.05 was accepted for statistical significance. 

Ethics

This research project was approved scientifically and ethically by the Başkent University Faculty of Medicine Research and Ethics Committee on 08/12/2020 (KA20/447).

## Results

Among the 1,091 (37.4%) patients, 802 had invasive ductal carcinoma (IDC; 73.5%), 78 had ductal carcinoma in situ (DCIS; 7.1%), 60 had invasive lobular carcinoma (ILC) (5.5%), 42 had invasive mixed carcinoma (IMC; 3.8%), and 109 had other cancer types (10.0%).

**Figure 1:**
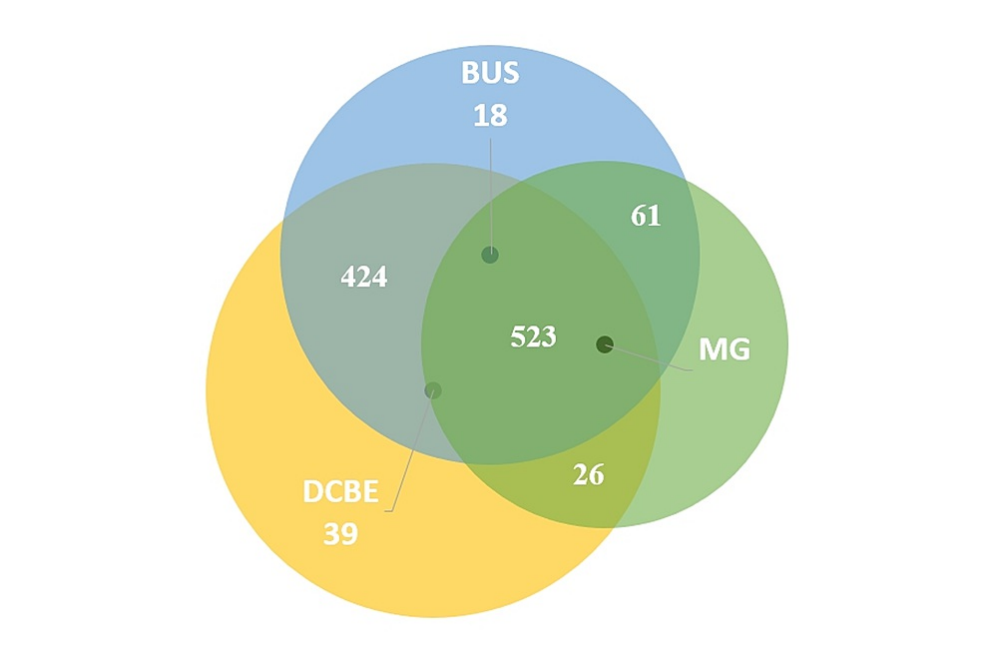
Proportion of cancers detected using diagnostic clinical breast examination, mammography, and breast ultrasonography DCBE: diagnostic clinical breast examination; MG: mammography; BUS: breast ultrasonography.

In all patients considered, cancer was detected (Figure 1) by MG, breast US and physician-performed DCBE in 47.9% (n = 523), by breast US and physician-performed DCBE in 38.9% (n = 424), by MG and breast US in 5.6% (n = 61), by physician-performed DCBE alone in 3.6% (n = 39), by MG and physician-performed DCBE in 2.4% (n = 26), and by breast US alone in 1.6% (n = 18).

The mean age of breast cancer diagnosis of the patients was 55.1 ± 13.5 years. Of the age range of the patients, 14.5% were under the age of 40 years, 24% were in the 40-49 years age group, 25.9% were in the 50-59 years age group, and 20.1% were in the 60-69 years age group (Table 1).

**Table 1:** Patient characteristics at the time of diagnosis according to histopathological types IDC: invasive ductal carcinoma; ILC: invasive lobular carcinoma; DCIS: ductal carcinoma in situ; IMC: invasive mixt carcinoma; OCS: oral contraceptives; HRT: hormone replacement therapy.

	Mean (standard deviation)					
	IDC	ILC	DCIS	IMC	Others	Total
Diagnosis age (years)	54.8 (13.1)	59.6 (14.8)	54.8 (14.3)	54.2 (16.1)	55.9 (14.3)	55.1 (13.5)
Menarche age (years)	13.1 (1.2)	13.1 (1.3)	13.1 (1.4)	13.2 (1.3)	13.0 (1.3)	13.1 (1.3)
Menopause (years)	47.8 (4.8)	48.3 (4.8)	48.5 (4.0)	47.2 (6.1)	47.7 (5.9)	47.9 (4.9)
Gravida	2.3 (1.7)	2.4 (1.4)	2.0 (1.3)	2.3 (1.9)	2.15 (1.6)	2.27 (1.7)
Parity	1.9 (1.4)	1.8 (0.9)	1.8 (1.2)	1.8 (1.4)	1.9 (1.5)	1.9 (1.3)
Abortion	0.2 (0.6)	0.2 (0.6)	0.1 (0.3)	0.1 (0.4)	0.1 (0.4)	0.2 (0.5)
Age at first birth (years)	24.6 (5.6)	27.0 (5.5)	25.0 (3.5)	23.5 (4.1)	25.3 (5.7)	24.8 (5.4)
Lactation length (months)	20.2 (23.7)	18.0 (13.4)	16.6 (21.5)	14.1 (17.7)	20.4 (24.1)	19.6 (22.9)
	n (%)					
Age groups (years)						
<40	112 (14.0)	5 (8.3)	13 (16.7)	12 (28.6)	16 (14.7)	158 (14.5)
40-49	201 (25.1)	13 (21.7)	17 (21.8)	4 (9.5)	27 (24.8)	262 (24.0)
50-59	210 (26.2)	15 (25.0)	22 (28.2)	10 (23.8)	26 (23.9)	283 (25.9)
60-69	163 (20.3)	13 (21.7)	13 (16.7)	9 (21.4)	21 (19.3)	219 (20.1)
70-79	82 (10.2)	7 (11.7)	10 (12.8)	5 (11.9)	10 (8.7)	114 (10.4)
≥80	34 (4.2)	7 (11.7)	3 (3.8)	2 (4.8)	9 (8.3)	55 (5.0)
Total	802 (73.5)	60 (5.5)	78 (7.1)	42 (3.8)	109 (10.0)	1.091 (100)
OCS/HRT	88 (11.0)	5 (8.3)	6 (7.7)	2 (4.8)	9 (8.3)	110 (10.1)
Benign breast diseases	71 (8.9)	3 (5.0)	15 (19.2)	5 (11.9)	8 (7.3)	102 (9.3)
Breast cancer history	4 (0.5)	0 (0.0)	1 (1.3)	0 (0.0)	3 (1.8)	7 (0.6)
Family history of breast cancer	187 (23.3)	18 (30.0)	21 (26.9)	4 (9.5)	19 (17.4)	249 (22.8)
Stages						
0			78 (100)		1 (0.9)	79 (7.2)
1	222 (27.7)	15 (25.0)		11 (26.2)	51 (46.8)	299 (27.5)
2	366 (45.6)	30 (50.0)		19 (45.2)	31 (28.4)	446 (40.9)
3	158 (19.7)	11 (18.3)		10 (23.8)	10 (9.2)	189 (17.3)
4	56 (7.0)	4 (6.7)		2 (4.8)	16 (14.7)	78 (7.1)

The mean age of menarche was 13.1 ± 1.3 years, and 5.9% of patients were below 12 years of age at menarche. Out of all the patients, 43.4% were in the post-menopausal period, and the mean age of menopause was 47.9 ± 4.9 years (min: 29, max: 61). Concerning birth, 14.1% of the patients were nulliparous, and 15.1% had never given a live birth. Regarding abortion, 8.1% of the patients had a history of medical abortion, and 12.3% had a history of abortion. The mean age of the first delivery of the patients was 24.8 ± 5.4 years (min: 15, max: 46). The average number of pregnancies was 2.3 ± 1.7 (min: 0, max: 14). The average lactation period was 19.6 ± 22.9 months. A history of OCS and/or HRT use was present in 10.1% (n = 110) of the participants. There was a family history of breast cancer in 22.8% (n = 249) of the patients (Table 1). Tumor, it is bilateral in 48.3% of the patients (n = 527), in the right breast in 46.1% (n = 503) and in 5.6% (n = 61).

The distribution of breast cancers by type was as follows: IDC in 73.5% (n = 802), DCIS in 7.1% (n = 78), ILC in 5.5% (n = 60), 3.8% (n = 42) IMC and 10.0% (n = 109) other histopathological types (Table 1). Of the patients participating in the study, 82.2% (n = 897) were positive for hormone receptor + (HR+), 16.1% (n = 176) for c-erb-B2 (HER2-neu), and 7.6% (n = 83) of the patients were diagnosed with triple-negative breast cancer.

The distribution of complaints was as follows; 64.5% (n = 704) breast mass, 11.9% (n = 130) pain, 4.0% (n = 44) nipple and/or skin retraction, 3.3% (n = 36) axillary mass, 3.1% (n = 34) nipple discharge, 2.1% (n = 23) open wound, 0.5% (n = 6) neck mass, and 0.3% (n = 3) upper arm swelling.

When DCBE findings were examined, the most common physical examination finding was a palpable breast mass (n = 941, 84.5%). Other findings were as follows: axillary lymphadenomegaly (n = 242, 21.7%), retraction (n = 140, 12.6%), nodularity (n = 123, 11.1%), operation scar (n = 54, 4%, 4%), discoloration (n = 41, 3.7%), edema (n = 40, 3.6%), ulceration (n = 35, 3.1%), tenderness (n = 12, 1.1%), and upper arm swelling (n = 5, 0.4%).

**Figure 2:**
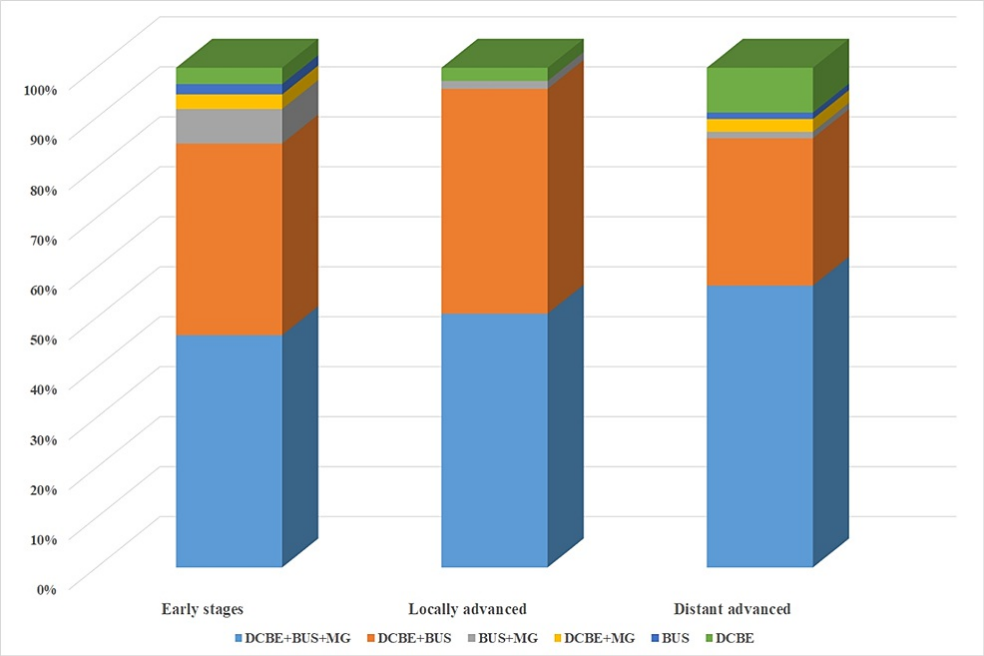
Distribution of diagnostic methods according to breast cancer stages DCBE: diagnostic clinical breast examination; MG: mammography; BUS: breast ultrasonography.

Distribution of cancers according to pathological stages: stage 0, 7.2% (n = 79); stage I, 27.5% (n = 299); stage II, 40.9% (n = 446); stage III, 17.3% (n = 189); and stage IV, 7.1% (n = 78). Early-stage breast cancer was present in 75.6% of the patients. Concerning detection of early-stage breast cancer, 73.2% (n = 383) of cancers were detected with DCBE, breast US, and MG, 74.6% (n = 316) of cancers were detected with DCBE and breast US, 93.4% (n = 57) of cancers detected with breast US and MG, 92.3% (n = 24) of cancers were detected with DCBE and MG, 94.4% (n = 17) of cancers were detected with breast US alone, and 69.2% (n = 27) of cancers were detected with DCBE alone (p = 0.00; Figure 2).

**Figure 3:**
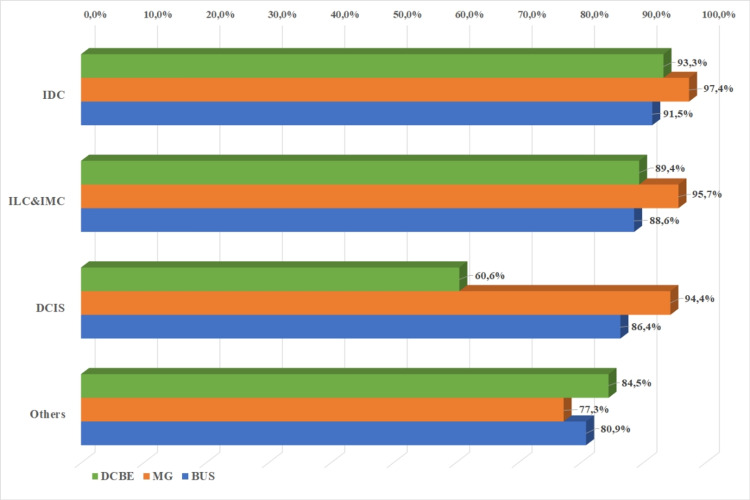
Distribution of diagnostic clinical breast examination, mammography, and breast ultrasonography positive results according to histopathological types IDC: invasive ductal carcinoma; ILC: invasive lobular carcinoma; IMC: invasive mixt carcinoma; DCIS: ductal carcinoma in situ; DCBE: diagnostic clinical breast examination; MG: mammography; BUS: breast ultrasonography.

Cancers (n = 58) with negative DCBE results were detected with MG in 89.8% (n = 52). Pathology was detected in 89.8% of breast cancers with DCBE. When examined according to histopathological diagnoses, DCBE was diagnosed in 93.3% of IDCs, 89.4% of ILCs and IMCs, and 84.5% of other histopathological types (p < 0.05). A total of 89.8% of cancers were detected with MG. When MG findings were examined according to histopathological types, 91.5% of IDCs, 88.6% of ILCs and IMCs, 86.4% of DCIS, 80% of other histopathological types, nine were detected with MG (p = 0.12). Breast US detected 95.1% of breast cancers, and 97.4% of IDCs, 95.7% of ILCs and IMCs, 94.4% of DCIS, and 77.3% of other histopathological types were detected with MG (p = 0.00; Figure 3).

The breast US result was positive in 84.3% (n = 86) of cancers with negative DCBE results. While 86.5% (n = 863) of the 998 cases with complete data for both DCBE and breast US were detected with both, 3.3% (n = 33) were detected with breast US alone, and 8.6% (n = 86) with DCBE alone. Both methods were negative in 1.6% of breast cancers. On the other hand, 67.3% (n = 33) of cancers with negative breast US results had positive DCBE results. While DCBE sensitivity was 89.8%, breast US sensitivity was 95.1% (p = 0.00; Table 2).

**Table 2:** Distribution of diagnostic clinical breast examination, mammography, and breast ultrasonography results in breast cancer

	Diagnostic clinical breast examination n (%)		Total
	Negative	Positive	
Mammography			
Negative	6 (1,2)	47 (9,0)	53 (10,2)
Positive	52 (10,0)	416 (89,8)	468 (89,8)
Total	58 (11,1)	463 (88,9)	521 (100,0)
Breast ultrasonography			
Negative	16 (1,6)	33 (3,3)	49 (4,9)
Positive	86 (8,6)	863 (86,5)	949 (95,1)
Total	102 (10,2)	896 (89,8)	998 (100,0)

## Discussion

In our study, the mean age of breast cancer diagnosis of the patients was 55.1 ± 13.5 years, with a median age of 54. However, in the study conducted by Özmen et al. by analyzing approximately 20,000 breast cancer patients between 2005 and 2017 in Turkey, the average age of breast cancer diagnosis was 51.8 and the median age was 50 [20]. According to the United States (US) Surveillance, Epidemiology, and End Results Program (SEER) 2013-2017 data, the median age for newly diagnosed female breast cancer in the US was 62 years [21]. According to these data, 10.2% of women diagnosed with breast cancer in the US were under 45, while those diagnosed under the age of 40 years in our study constituted 14.5%. According to Global Cancer Observatory (GLOBOCAN) 2020 data, 29% of those diagnosed with breast cancer worldwide were women under the age of 50 years, while women under 50 years constituted 40% of those diagnosed with breast cancer in Turkey [4]. Similarly, in our study, 38.5% of the cases were diagnosed with breast cancer under the age of 50 years, and breast cancer tended to be seen in our country at a younger age than the world average. 

In our study, 85.6% of the patients diagnosed with breast cancer consulted a physician with a symptom in the breast. Similarly, in the study of Özmen et al., 86% of the women applied to a physician with a symptom in the breast and 14% without any complaints [20].

The diagnosis of 47.9% (n = 523) of the patients participating in our study was through DCBE, breast US, and MG; the diagnosis of 38.9% (n = 424) was through DCBE and breast US; 5.6% (n = 61) were diagnosed through breast US and MG; 2.4% (n = 26) were diagnosed through DCBE and MG; 1.6% (n = 18) of them was diagnosed through breast US alone; and 3.6% (n = 39) of them were diagnosed through DCBE alone. The fact that breast US sensitivity with MG was lower than breast US sensitivity with CBE was contrary to the general literature. We think that the main reason for this is the relatively high rate of dense breast parenchyma in the patient population. Findings reported in studies that MG sensitivity is considerably reduced in the presence of dense breast parenchyma support this idea [22]. In addition, while almost the entire study population had breast US information, the group with MG information was almost half of this number. Also, this situation can be considered as a negative limitation; 73.2% (n = 383) of cancers detected by DCBE, breast US and MG were early-stage breast cancer; 74.6% (n = 316) of cancers detected by DCBE and breast the US, 93.4% of cancers detected by breast US and MG (n = 57), 92.3% (n = 24) of cancers detected with DCBE and MG were early-stage breast cancer; 94.4% (n = 17) of those detected with breast US only, 69% of those detected with DCBE only, 2 (n = 27) were early-stage breast cancer (p < 0.05). Significantly less early-stage breast cancer was detected with only DCBE than with other methods or combinations of methods (p < 0.05).

In our study, the sensitivity of DCBE was found to be 88.9%. Of cancers detected by DCBE, 5.1% (n = 57) were stage 0, 26.3% (n = 293) were stage I, 42.5% (n = 473) were stage II, 18.4% (n = 205) were stage III, and 7.6% (n = 85) were stage IV breast cancer.

In our study, MG sensitivity was found to be 89.8%, and there was no significant difference between histopathological types in terms of MG sensitivity. MG sensitivity for breast cancer varies between 70 and 90% in the literature, but this rate shows a significant decrease in women with dense breast parenchyma [11]. In our study, dense breast parenchyma was found in 52.1% (n = 449) women with MG data. Dense breast parenchyma was present in women diagnosed with stage 0 (DCIS) breast cancer most frequently (61.1%). In a study similar to ours conducted on 66,680 women, dense breast parenchyma was found in 47.9%. MG sensitivity was found in 61.5% of these women and 86.6% in women without dense breast parenchyma [22]. In a study conducted on women with DCIS, the rate of women with dense breast tissue was found to be 58%, and the rate of these women being younger, pre-menopausal and Asian was found to be higher [23]. This explains the high prevalence of dense breast tissue in women with breast cancer in a country like Turkey, where the incidence of breast cancer at a young age is higher than the world average.

Breast US was able to detect 95.1% of breast cancers in our study. In a meta-analysis, breast US sensitivity was found to be 89.2% in low- and middle-income countries [24]. In the study conducted by Houssami et al., the sensitivity of breast US was 13.2% higher than MG in women younger than 45 years of age [25]. Since the incidence of breast cancer in young women in our study was higher than the world average, the sensitivity of breast US was higher than MG, as in the study of Houssami et al. When examined according to histopathological types, breast US sensitivity was found the most in IDC (97.4%). However, ILC + IMC had similar sensitivity in the diagnosis of DCIS (95.7%, 94.4%). The most common finding in breast US is a hypoechoic nodular lesion with irregular borders, similar to the one frequently described in the literature [26].

While 89.8% (n = 416) of 521 cases with MG and DCBE data were detected with both MG and DCBE, 10.0% (n = 52) were detected only with MG, and 9% (n = 47) with DCBE only. No diagnosis was possible with either method for 1.2% (n = 6). According to these data, 89.8% (n = 52) of cancers (n = 58) that DCBE could not detect were detected by MG, and 88.7% (n = 47) of cancers that could not be detected by MG (n = 53) were detected by DCBE. The sensitivity of DCBE and MG in breast cancer diagnosis was similar (88.9%, 89.8%, p >0.05). According to our study, 9.0% (n = 47) of breast cancers would have been overlooked if DCBE had not been performed. Similarly, in a study conducted in 2016, it was reported that 8.7% of breast cancers were detected by CBE alone and would have been overlooked if MG alone had been performed [11]. There are also studies in which palpable cancers are missed at a rate of 10-17% in women undergoing MG screening [16,27,28]. In addition, in a study conducted in Japan, the contribution of CBE between the ages of 60-70 years could be neglected as it was minimal (1.0-2.7%), but for the early diagnosis of breast cancer in people outside this age group, the percentage of CBE to MG was found. Therefore, it has been suggested that it contributes 3.3-7.7% and that CBE should be added [29]. One of the important issues emphasized in the light of all these data is that CBE should be performed before imaging methods in the presence of any symptoms in the breast. This is because it was found in a study that approximately 11% of the patients presenting with the complaint of a breast mass and 4% of the patients presenting with any symptom, including the mass, had breast cancer [30]. As a matter of fact, the fact that CBE detected approximately 7-17% of tumors in various studies, including our study, clearly demonstrates the importance of this situation [11,16,27-29].

While 86.5% (n = 863) of the 998 cases with breast US and DCBE data were detected with both breast US and DCBE, 3.3% (n = 33) were detected only by breast US, and 8.6% (n = 86) were detected only by DCBE. Neither method could diagnose cancer in 1.6% (n = 16) of the cases. According to these data, 84.3% (n = 86) of cancers (n = 102) that DCBE could not detect were detected by breast US, and 67.3% (n = 33) of cancers (n = 49) that breast US could not detect could be detected by DCBE. While the sensitivity of DCBE was 89.8%, the sensitivity of breast US was 95.1%, and there was a statistically significant difference between them (p = 0.000). Breast US is the primary diagnostic method used in the differentiation of cystic and solid lesions. It is also superior to MG in the diagnosis of breast cancer in women with dense breast parenchyma. As stated in our study, dense breast parenchyma was detected in approximately half of the cases. In this case, it is also usual to find the sensitivity of breast US higher than MG and CBE, which is found to have similar sensitivity with MG.

There were some limitations in our retrospective study, which was limited to the variables recorded in the database performed in a tertiary healthcare institution. We should consider that we can obtain different results with a sample from the general population due to the use of records of applications belonging to only a hospital. Since the hospital, we work in is a tertiary hospital, the incoming patients have probably applied by referral. This possibility may have led the patients included in the analysis to represent a biased selection from the general population. It may also be the reason for the high rate of missing breast examination data (60%) in all records. Therefore, this study cannot provide data on breast examinations performed by primary and secondary care physicians.

All patients with appropriate patient records were included without randomization for the study group. The fact that the patient file should be complete may have caused a bias.

Although a mass in breast cancer is a finding that can develop over the years, it is the main examination finding of the patients. This finding may be because the study was conducted with the records of a tertiary health institution, as we mentioned. About 60% of the total patient group reached was excluded from the analysis. The main reason for this is the incomplete examination record in the patient's file. This may be due to the laziness of the physicians in recording the examination findings, and in addition, perhaps the patient does not have any abnormal examination findings. If the latter situation is valid, leaving 60% of the total sample out, for this reason, can also have negative consequences for CBE. It is impossible to exclude the possibility that other methods made such a large proportion of breast cancer diagnoses without examination findings. Therefore, the inability to detect whether these patients were diagnosed with breast cancer by another method without any findings on examination was an important limitation.

Data were obtained from a single-center, and MG and breast US data were obtained from radiologists' reports with 4-15 years of experience. DCBE was performed by general surgery specialists with 5-22 years of experience. This should be taken into account, as the sensitivity of DCBE can vary greatly depending on the experience of the practicing physician. Since We did not record pre-menopausal or post-menopausal information in most patients in the database, We could not make an effective investigation in our study. In addition, the sensitivity of the diagnostic methods was not examined according to the dense breast parenchyma and by classifying them according to age. Therefore, although this study determined the sensitivity of DCBE, We could not examine the false positive rate in breast cancer diagnosis and its effect on mortality.

In our retrospective study, although we detected the sensitivity of DCBE in the diagnosis of breast cancer, the specificity, false positivity, and negativity rates of DCBE were not determined due to the inconsistency of the study design. In many studies, it has been concluded that CBE should be decided on a community basis, as it may cause an increase in unnecessary referral and biopsy rates.

As a result, in societies where screening participation and awareness of breast cancer are low, a breast cancer diagnosis is made after the complaint of a palpable mass in the breast, and breast cancer is seen at a more advanced stage. Younger ages, breast self-examination (BSE), and CBE still maintain their importance. CBE is a method of diagnosing breast cancer that prevents cancer from being missed at a significant rate and is very cost-effective. Family physicians should educate women in the population they are responsible for in terms of BSE, referring them to higher-level treatment institutions in case of detection of pathology by performing CBE when necessary, educating them about screening programs according to age groups, and directing them for screening.

## Conclusions

Early diagnosis of breast cancer, the most common malignancy in women, and increasing the success of treatment are of great importance in public health and effective use of health resources. 

In societies where participation in breast cancer screening and breast cancer awareness is low, a breast cancer diagnosis is seen when the cancer is in more advanced stages. Also, for countries where breast cancers are observed at younger ages, SCBE and CBE still maintain their importance. With its sufficient sensitivity, CBE is among the important diagnostic methods that prevent breast cancer from being overlooked, especially in cases where health resources are limited, and access to mammography and breast ultrasonography is limited.
